# COVID-19: A Driver for Disruptive Innovation of the Emergency Medicine Residency Application Process: Recommendations from the Council of Residency Directors Application Process Improvement Committee

**DOI:** 10.5811/westjem.2020.8.48234

**Published:** 2020-08-19

**Authors:** Alexis Pelletier-Bui, Doug Franzen, Liza Smith, Laura Hopson, Lucienne Lutfy-Clayton, Kendra Parekh, Mark Olaf, Tom Morrissey, David Gordon, Erin McDonough, Benjamin H. Schnapp, Mary Ann Edens, Michael Kiemeney

**Affiliations:** * Cooper Medical School of Rowan University, Department of Emergency Medicine, Camden, New Jersey; † University of Washington School of Medicine, Department of Emergency Medicine, Seattle, Washington; ‡ University of Massachusetts Medical School Baystate Health, Department of Emergency Medicine, Springfield, Massachusetts; § University of Michigan Medical School, Department of Emergency Medicine, Ann Arbor, Michigan; ¶ Vanderbilt University School of Medicine, Department of Emergency Medicine, Nashville, Tennessee; || Geisinger Commonwealth School of Medicine, Department of Emergency Medicine, Scranton, Pennsylvania; # University of Florida College of Medicine-Jacksonville, Department of Emergency Medicine, Jacksonville, Florida; ** Duke University, Division of Emergency Medicine, Durham, North Carolina; †† University of Cincinnati College of Medicine, Department of Emergency Medicine, Cincinnati, Ohio; ‡‡ University of Wisconsin School of Medicine and Public Health, BerbeeWalsh Department of Emergency Medicine, Madison, Wisconsin; §§ Louisiana State University Health Shreveport, Department of Emergency Medicine, Shreveport, Louisiana; ¶¶ Loma Linda University School of Medicine, Department of Emergency Medicine, Loma Linda, California

## Abstract

The coronavirus disease (COVID-19) pandemic has had a significant impact on undergraduate medical education with limitation of patient care activities and disruption to medical licensing examinations. In an effort to promote both safety and equity, the emergency medicine (EM) community has recommended no away rotations for EM applicants and entirely virtual interviews during this year’s residency application cycle. These changes affect the components of the EM residency application most highly regarded by program directors – Standardized Letters of Evaluation from EM rotations, board scores, and interactions during the interview. The Council of Residency Directors in Emergency Medicine Application Process Improvement Committee suggests solutions not only for the upcoming year but also to address longstanding difficulties within the process, encouraging residency programs to leverage these challenges as an opportunity for disruptive innovation.

## INTRODUCTION

The coronavirus pandemic has substantially disrupted undergraduate medical education. While creative solutions have been implemented for classroom-based activities, suspension of patient contact^1^ causes a disproportionate impact on students in clinical rotations. Rotational experiences are critical to the development of learners into independent practitioners and comprise an important component of the residency selection process.^2^ Emergency medicine (EM) is particularly vulnerable to the impact of the altered clinical learning environment as emergency departments (ED) are dealing with large volumes of coronavirus disease (COVID-19) patients and often low volumes of non-COVID patients.^3^ Further, EM courses are frequently only available to senior medical students, limiting students’ opportunities for exposure to the specialty.^4^

Given these constraints, residency programs will need to adjust their expectations for residency application materials, including numbers and types of recommendation letters, clinical experiences, and United States Medical Licensing Examination (USMLE) scores. Considering forecasts for a second surge of coronavirus in the fall or winter, paired with ongoing or reinstituted travel restrictions, the Coalition for Physician Accountability (Coalition) has recommended that all residency programs commit to virtual interviews for the entire upcoming application cycle, and the EM community has released a statement supporting this recommendation.^5^,^6^ While challenging, these changes do, however, provide an opportunity to explore alternative models for recruiting the next generation of emergency physicians while simultaneously identifying creative solutions to longstanding difficulties such as volume of applications and cost. We encourage programs to respond to the challenges presented by COVID-19 not just reactively, but with an eye toward transformative change of the application and interview process. We offer here suggestions on the application and interview process, many of which could be carried forward into future application cycles.

## APPLICATION REVIEW

### EM Rotations and Letters of Recommendation

#### Challenges

EM program directors (PD) cite the Standardized Letter of Evaluation (SLOE) as the most important component of the residency application when making interview invitation decisions.^2^,^7^–^9^ Many residency programs expect two SLOEs prior to making interview offers.^10^ Academic group SLOEs from residency programs carry the most weight,^8^,^9^ placing a greater burden on applicants from schools without a home EM residency (“orphan” students). Recognizing that evaluations from EM subspecialty rotations or EM faculty not affiliated with a residency program also have merit, the Council of Residency Directors in EM (CORD) has developed modified SLOEs (https://www.cordem.org/resources/residency-management/sloe/), which carry less weight.^9^ EM residency programs place lowest value on letters of recommendation from non-EM faculty.^9^

With clinical rotations suspended nationally, formerly predictable clerkship curricula are in flux, potentially delaying fourth-year rotations, including EM. Even those who are able to resume regularly scheduled fourth-year rotations are discouraged from performing away rotations to promote equity between applicants;^5^,^6^ therefore, the majority of applicants will only have one traditional SLOE in their residency application. At an even greater disadvantage are “orphan” applicants, who may have difficulty obtaining even a single EM rotation. Applicant groups that are disproportionately affected by “orphan” status are osteopathic and international medical graduate (IMG) applicants. Restricted access to fourth-year EM rotations may also impact the number of applicants to EM, as up to 36% of surveyed US medical student applicants to EM didn’t decide until their fourth-year of medical school.^11^ On the other hand, some students in a similar position may still attempt to pursue a career in EM only to develop specialty regret and potentially leave an unfilled position in a residency program.

#### Suggested Solutions

Decreased rotation availability and fewer EM residency-authored SLOEs will require rethinking the current hierarchy of letters and giving more value not only to non-traditional SLOEs, but also to letters authored by non-EM faculty. To increase efficacy and make the task easier for non-EM letter writers, CORD has assembled a committee to develop a template for writers, highlighting the attributes EM PDs specifically look for in a letter and in an applicant. This template, termed the “O-SLOE” for “off-service” or “other rotation,” is now available on CORD’s SLOE webpage (https://www.cordem.org/resources/residency-management/sloe/). This can be distributed to medical school deans and clerkship director organizations. Continued use of this template beyond this extraordinary academic year could increase the utility and rigor of non-EM evaluations in future application cycles. This same committee has updated the EM-faculty SLOE to allow writers to detail how COVID-19 has affected their student rotations (“on the reCORD,” CORD listserv communication, June 23, 2020).

The Coalition and EM community recommend that away rotations be discouraged for the 2020–2021 academic year with the exception of “learners who have a specialty interest and do not have access to a clinical experience with a residency program in that specialty in their school’s system,” and “learners for whom an away rotation is required for graduation or accreditation requirements.”^5^,^6^ Given there are over 80 identified schools without an established EM residency program and there is a possibility of students being limited from performing an EM rotation at their own institution due to high volume of COVID-19 patients (Susana Tsao, DO, CORD listserv communication, May 4, 2020), we support these exceptions to the “no aways” policy. We also support the recommendation for institutions that are still hosting rotators to preferentially accept students who are unable to obtain a SLOE from their home institution.^12^,^13^ A live document of schools without a home EM residency program can be found here (https://bit.ly/37UKYEp).

Proactive efforts should be made to increase EM rotation availability for EM-bound students. Potential strategies include shortening the length of the rotation (eg, from four weeks to three weeks) or reducing the number of shifts required per rotator, allowing more rotators per block. The timeframe for completion of EM rotations could be expanded beyond the traditional summer months, particularly considering the recently updated Electronic Residency Application Service (ERAS) application timeline with residency programs not being able to view residency applications until October 21, 2020.^14^

Even with utilization of these strategies, some EM-bound applicants may still not have a SLOE in time for file review. In such cases, we support the recommendation that an EM advisor write a letter of recommendation specifically incorporating the key elements of the SLOE.^13^ It should explicitly state that the applicant was unable to obtain a SLOE in time for file review secondary to COVID-19 and vouch for the applicant’s desire to pursue a career in EM, their career decision process, and potential for success. Additional insight into an applicant’s prior EM experiences beyond what can be gleaned from their curriculum vitae could also be helpful.

### Board Scores

#### Challenges

After SLOEs, the USMLE Step 1 and Step 2 Clinical Knowledge (CK) exams are the next biggest factors of importance in selecting applicants for interviews.^2^ In the 2018 National Resident Matching Program PD survey, 48% of PDs required USMLE Step 2 CK and 31% used a target score for Step 2 CK when considering applicants for interviews.

Most Prometric Testing centers, which administer the USMLE Step 1 and 2 CK, were closed until June 1, 2020, and are still not running at full capacity based on governmental ordinances and advice from the Centers for Disease Control and Prevention and the World Health Organization.^15^ Gradual and incomplete opening, paired with the backlog of individuals competing for standardized testing, has lead to delays in testing and will likely result in later release of scores for many applicants. Likewise, USMLE Step 2 Clinical Skills (CS) testing has been suspended for 12–18 months.^16^ IMG applicants may be disproportionately affected by the inability to complete Step 2 CS, previously a requirement for certification by the Educational Commission for Foreign Medical Graduates (ECFMG).^17^ PDs may not have the full complement of USMLE scores available to them that they have traditionally relied upon for applicant screening and rank list submission. Additionally, it is unknown how these delays will affect applicants’ ability to obtain USMLE scores in time for medical school graduation.

#### Suggested Solutions

There are potential solutions to compensate for the testing bottleneck. Six US medical schools have opened regional testing centers to allow for additional testing options outside of Prometric and the USMLE is working with eligible medical schools across the US to host one-day testing events to administer Step 1 and Step 2 CK in the near future.^18^. The ECFMG has created five new pathways to meet the requirements for ECFMG certification for those IMG applicants who have yet to complete Step 2 CS for the 2021 match cycle.^17^ We encourage programs to consider these pathways as a substitute for the Step 2 CS examination. Programs that previously required Step 2 CK or CS for interview or ranking should consider temporarily revising their approach and policies. Institutional expectations or state-level requirements for licensure should be clearly articulated and communicated to allow applicants to make educated application decisions.

However, we encourage residency programs to use this opportunity to take a new approach to the USMLE. Quantitative metrics such as USMLE Step 1 and 2 CK are commonly used to stratify and filter students. While scores do have some correlation with the likelihood of passing the American Board of Emergency Medicine qualifying exam,^19^ these scores do not correlate with clinical proficiency or success in EM residency.^20^ Reducing the influence of USMLE scores when screening applicants has long been discussed among EM educators, with a goal to transition toward more holistic application review. The transition of USMLE Step 1 to a pass/fail score within the next few years,^21^ combined with the testing disruptions of the COVID-19 pandemic, present an opportunity for more rapid change.

Holistic application review involves programs performing honest self-assessment and appraisal of residency graduates to determine which character traits and attitudes are valued and associated with success in their program and then seeking out applicants with those qualities.^22^ Combining holistic application review with an understanding of a program’s own strengths and challenges in resident development can help programs identify and recruit applicants who are more likely to match successfully with them and succeed in training.

### Medical Student Performance Evaluation (MSPE)

#### Challenges

The MSPE is traditionally released at the beginning of October, marking the unofficial start of interview offers to applicants. Programs often wait for the MSPE before sending interview invitations, as it may describe professionalism or academic concerns that do not appear elsewhere in the application. With fewer SLOEs to review, and varying clinical experiences between students, the MSPE may take on additional importance this year. As a result of cancelled clinical rotations, some applicants may not have completed the core clerkships that traditionally contribute significantly to the MSPE.

#### Suggested Solutions

ERAS has amended the residency application timeline to allow for MSPE release on October 21, the same day that residency programs will be able to begin reviewing applications.^14^ Delaying these components of the application cycle will effectively push back the start of interview season and might relax the time pressure on students, schools, and programs.

Advising students to complete most, if not all, core clerkships before the release of the MSPE will offer applicants a better chance at receiving interview offers than if some core experiences are incomplete and updated at a later time. If not possible, a mechanism to allow regular updates to the MSPE may be useful.

COVID-19 has not affected geographic regions equally. Programs will look to the MSPE to delineate the pandemic’s effect on the learning environment. We suggest adding a standardized “pandemic response” section to MSPEs. Schools should describe how they adapted, including what dates students were excluded from clinical experiences, what clerkship experiences may have been virtual, and any policies that prohibited students from seeking away rotations. Additionally, any action by students who went above and beyond to help during this time of crisis could be very useful information to EM PDs. If all schools use the MSPE to outline a school’s pandemic response, readers will be able to place a student’s record into context and identify outliers within a single school or between schools.

### Numbers of Applicants/Applications

#### Challenges

Cancellation of EM away rotations and delays in senior electives while students complete core clerkships may decrease the total number of EM applicants, due to decreased exposure to EM and uncertainty about the specialty. Simultaneously, the number of applications per applicant may increase due to perceived deficiencies (eg, lack of SLOEs and/or USMLE scores, atypical MSPE) and the inability to hone residency and geographic preferences via audition rotations.

#### Suggested Solutions

While we cannot control students’ reactions to the uncertainty of this application cycle, the EM community can make efforts to mitigate other anxiety-provoking elements of the application process, foremost a lack of transparency. Clear communication will be critical, including informing students when they can expect to hear about interview offers, a program’s preferred method of contact for questions or updates, and clear expectations for wait-list status.

Programs might consider allowing students to submit an optional statement of purpose or intent, in which students have the opportunity to communicate a particular interest in a given program or region. Allowing students to communicate what they perceive to be their “fit” with a program can help application reviewers identify students who are likely to be high-yield candidates and successful matches. This was instituted by the otolaryngology community as a requirement in the 2015–16 application cycle as a program-specific paragraph added to the end of an applicant’s personal statement. While this measure was found to be effective in decreasing the overall number of applications per applicant, it is also thought to have made otolaryngology appear less welcoming to medical students as there was an overall decline in the number of medical students applying to otolaryngology after this initiative. As a result, the Otolaryngology Program Directors Organization has now made the program-specific paragraph optional.^23^,^24^ Programs that wish to institute a program-specific paragraph as part of their application process are asked to publish these requests on ERAS when registering in ERAS Account Maintenance. This information will then be displayed to applicants as they research programs (Elise Lovell, MD, on behalf of Amy Mathis, Senior Director of ERAS, CORD listserv communication, May 11, 2020).

One low-cost, low-effort method for students and PDs that may allow PDs to identify high-yield candidates amidst increasing application numbers is the institution of preference signaling, or a “star” system, which gives students a limited number of “stars” to allocate to their most desired programs. This method is used in other professions and has been proposed for use in orthopedics and otolaryngology.^25^–^28^ A computer-simulated model using otolaryngology match data found that applicants voluntarily adding preference data to their application enhanced the practical number of interview invitations for all applicants and could potentially allow more holistic file review of high-yield candidates to their program.^28^ However, implementation would require the collaboration with the Association of American Medical Colleges (AAMC) and development of technology that may be challenging to institute in time for this year’s residency application cycle.

## INTERVIEWS

### Challenges

The interview and surrounding interactions are routinely cited by PDs as a major factor in ranking decisions.^2^ The Coalition recommends that all residency programs commit to virtual interviews for the upcoming application cycle.^5^ Opportunities for social events and “second looks” may also be curtailed. These interactions play key roles in assessing goodness of fit, both from the applicant’s and the program’s perspective, and are cited as particularly important for ranking decisions by under-represented minorities in medicine.^29^

### Suggested Solutions

#### Video Interviewing

CORD supports replacing traditional in-person interviews with video interviews to try to maintain an equitable interview process for applicants and programs through this entire residency application cycle.^6^ Several programs have successfully demonstrated high satisfaction rates with video interviews, highlighting the advantages of reducing time and cost burdens for both the applicant and programs.^30^–^34^ Video interviews have the potential to level the playing field for applicants of low socioeconomic status who may not have the financial ability to travel for interviews.^30^,^31^. Reduced absences from clinical rotations would further enhance education in the fourth year of medical school.^34^

Despite some of the intrinsic appeal of video interviews,^30^,^31^ there are limitations compared to traditional on-site visits. Applicants face challenges accurately representing who they are as people, or in providing comfort in ranking a residency program,^30^,^32^ as many subtle indicators occur outside the actual interview discussion. Other perceived disadvantages are the inability to learn about a city or program and difficulty interacting with current residents and faculty.^31^ One study showed that neither applicants nor interviewers were comfortable making video interviewing the only means of interviewing,^32^ although another showed that some applicants prefer an initial video interview with the ability to later visit a program.^31^ This may be an option for some programs if travel restrictions and social distancing are in effect in the fall but later lifted prior to rank list submission.

For recommendations on the mechanics of conducting virtual interviews, consider reviewing the tips for PDs and interviewers published by the AAMC and the Compendium of Resources published by the Coalition.^35^–^37^

#### Highlighting the Program Beyond the Interview

We must prepare for no in-person visits to residency programs. While this may offer challenges in showcasing a residency program, this is not insurmountable.

Residencies may find it beneficial to leverage existing experiences taking place at their institutions, rather than creating entirely new content for applicant consumption. For example, video of residents interacting with faculty during a small group session may provide invaluable information to applicants about didactic quality, how faculty and residents interact, and resident camaraderie.

If not already available, programs should consider creation of expanded content (written, photographic, video) that highlights their program’s goals, strengths, and educational philosophy as well as what they are looking for in an applicant. New content will likely need to be created to replace the tour of the ED, resident spaces, the hospital and the surrounding geographic region. While this runs the risk of advantaging programs with the time and resources to produce professional-appearing content, these costs likely pale in comparison to that of running full interview days, and the potential advantages to program and applicants in terms of increased information to make their residency decisions may outweigh these risks.

Wide distribution of content will be key. An institution residency webpage, an external website, and social media accounts, if allowed by the institution, will likely be the best options for highlighting this material. Video content could also be uploaded onto YouTube or Vimeo. Given the high utilization of the Emergency Medicine Residents’ Association (EMRA) Match website by medical students, programs should ensure that their webpages and social media sites are updated on their EMRA Match profile.

#### Helping Applicants get a “Feel” for a Program and Assess for Fit

Programs could consider hosting mini virtual-EM rotations with the ability for students to “attend” a short series of video didactic conferences at outside sites. This could be particularly beneficial for programs that traditionally depend on their EM rotation experience as a recruitment tool for outside rotators. Benefits to the student include the abilities to get a glimpse of a program’s teaching styles, facilitate interaction with faculty and residents at other EM programs beyond a single interview day, and make up for lost educational opportunities due to the reduction of the typical number of EM rotations. Virtual rotations could be particularly beneficial for IMG applicants who will likely have difficulty obtaining even one EM rotation due to travel restrictions. We encourage institutions hosting virtual EM rotations to strongly consider accepting IMG students into their rotation to help balance this inequity. To ensure a good ratio of faculty to learners, students should only be able to participate in a small number of these, equivalent to the number of away rotations typically performed. Students’ selections of which of these experiences to participate in could give programs insight into what type of program (or where) the student is ideally looking to match.

For students who are invited to interview with a program, an invitation could be extended to “attend” conferences by sharing a virtual forum link. Asking selected faculty or residents to remain online with the students after the conference could be another way for applicants and faculty/residents to get to know each other.

Programs could “host” online pre-interview socials or lunch-time “hangouts” with residents, with the ability to break up into smaller rooms or even one-on-one conversations. Programs could also consider hosting a “hangout” in the spring or summer to generate interest in their program, and record/post it for interested applicants to review. A topic for consideration during these “hangouts” is the kind of resident that really shines in their program, and what kind of applicant might have struggles or find the environment less palatable. This might help attract best-fit applicants.

Programs could also consider using a tool that allows interviewees to guide their interview in a way that is meaningful for them and allows a more accurate impression of themselves compared with a traditional interview, as used by one otolaryngology residency program.^38^ The tablet-based interactive Candidate Assessment Tool allows residency applicants to select questions via a homepage of prerecorded video clips from key leaders in the institution, covering a variety of topics, interests and Accreditation Council for Graduate Medical Education core competencies.

## EXPLORATION OF NEW METHODS/METRICS FOR APPLICANT ASSESSMENT

### Challenge

With the potential disruption of metrics and methods that have traditionally been highly valued in the residency selection process,^2^ programs will have challenges identifying and stratifying applicants who may be successful in their residency program. The combination of this pandemic and USMLE Step 1 moving to pass/fail creates an opportunity for graduate medical education (GME) to develop and explore new methods to better holistically review applications. Another goal of many EM residency programs is to increase the number of under-represented minorities in one’s program, as evidenced by the surge in under-represented minority EM clerkship scholarships.^39^

### Suggested Solutions

While the EM community was not interested in continuing the AAMC Standardized Video Interview pilot as a new metric for EM residency selection,^40^ there may be alternative tools that we can explore to help identify successful applicants to our EM residency programs. Pre-hire assessments are used by eight of the top 10 US private employers and by 57% of large US employers.^41^ Some of these assessments have demonstrated utility in undergraduate medical education and GME as well.^42^–^48^

For example, emotional intelligence testing has been shown to have positive correlation with medical school success.^43^ Personality testing to assess for fit is widely used in other industries and some data indicate that it may be effective for residency selection as well.^42^ Situational judgment testing through methods such as the Computer-based Assessment for Sampling Personal Characteristics (CASPer), has demonstrated moderate predictive validity to national licensure outcomes in Canada^44^ and is required by all medical school applicants in Canada and two US medical schools.^45^ Its utilization in general surgery has been associated with overall performance in residency,^46^ allowed for more general surgery interview offers to under-represented minorities in medicine,^48^ and did not detract applicants from applying to the general surgery programs that implemented its use.^45^,^48^

While it is neither feasible, nor advisable, to incorporate these assessments for all EM applicants during this application cycle, now is a better time than ever to begin exploring and validating these, or other methods, for potential future use in the EM residency application process. Altus Assessments, the developer of CASPer, and the National Board of Medical Examiners are collaborating to explore the use of CASPer during this residency application season (Elise Lovell, MD, on behalf of Altus Assessments and the National Board of Medical Examiners, CORD listserv communication, June 21, 2020). Programs interested in participating in this research project can fill out this form (http://bitly.ws/9s73).

## CONCLUSION

While COVID-19 presents significant challenges to medical education and the residency application process, the GME community can view this pandemic as an opportunity to explore disruptive change. Just as quarantine orders eliminated barriers to virtual meetings and appointments overnight, we have the chance to make important changes to the residency application process that will benefit programs and applicants for years to come (Tables 1 and 2). We should embrace this opportunity while simultaneously working to preserve the essential components of the process. Only time will tell how the pandemic will influence the match process, and what adaptations will be most helpful. The CORD Application Process Improvement Committee is committed to making the most of the situation and plans to follow up with a report detailing the impacts and best practices for addressing similar challenges in the future.

## Figures and Tables

**Table 1:** Suggested solutions to address challenges in the emergency medicine residency application review process amidst COVID-19.

Challenge	Suggested solutions
Emergency medicine rotations and letters of recommendation	Strategies to increase applicants’ exposure to EM: Increase the number of EM rotators through an institution by shortening rotation length or decreasing required number of shifts. Expand time frame for away rotators to complete EM rotation beyond traditional summer months. Support one EM rotation for all by prohibiting away rotations for applicants with a home residency program and reserving away rotation slots for applicants without access to a Standardized Letter of Evaluation (SLOE) from their home institution. If a student is unable to obtain a SLOE, have an advisor write a letter incorporating the key elements of the SLOE. Provide more weight to non-residency affiliated EM faculty SLOEs, EM sub-specialty SLOEs, and letters from outside of EM. Encourage use of the O-SLOE (for off-service or other rotations) template for non-EM physician letter writers, which details characteristics that are valued by EM program directors (PDs).
Board scores	Consider the new certification pathways instituted by the Educational Commission for Foreign Medical Graduates as a substitute to the Step 2 Clinical Skills (CS) exam for international medical graduates Consider revision of policies requiring Step 2 Clinical Knowledge and CS for interview offer and/or ranking. Engage in holistic application review.
Medical Student Performance Evaluation (MSPE)	MSPE release and residency application availability to PDs has been delayed to October 21, relaxing the time pressure on students, schools and programs. For applicants who were still unable to complete core clerkships in time for MSPE release, schools can consider allowing amendments to the MSPE. Schools should outline their pandemic response in their MSPE, including how their students’ clinical experiences were affected by the pandemic and any prohibitions in obtaining EM rotations.
Number of applicants/applications	Programs should be transparent on their websites with regard to expectations, requirements and timelines. Consider allowing students to submit an optional statement of interest to specific programs within their personal statement. Communicate this desire/expectation with the Electronic Residency Application Service (ERAS). Consider instituting preference signaling in ERAS where applicants can designate their top residency choices during application.

*EM*, emergency medicine; *PD*, program director.

**Table 2:** Suggested solutions to address challenges in the emergency medicine residency interview process amidst COVID-19.

Challenges	Suggested solutions
In-person interviewing	Replace with video interviews. Augment video interviews with an option to visit the program at a later date pending travel and social distancing restrictions. For recommendations on the mechanics of conducting virtual interviews, consider reviewing the tips for program directors and interviewers published by the Association of American Medical Colleges and the Compendium of Resources published by the Coalition of Physician Accountability.
Highlighting the program beyond the interview	Record and post videos of existing resident experiences, didactics, etc. Create expanded content (written, photographic, video) with particular attention to replacing the traditional tour of the facility, resident spaces and geographic area. Promote content via institution website and social media (Twitter, Instagram, Facebook, YouTube). Ensure Emergency Medicine Residents’ Association Match profile and social media links are up to date.
Helping applicants get a “feel” for a program and assess for “fit”	Host a mini virtual EM rotation. Invite applicants to “attend” conference virtually. Host online pre-interview and lunch socials. Host resident “hang outs” with residents fielding applicant questions about what kind of applicant shines in their program. Use an interactive interview tool that allows applicants to guide their interview in a way that is meaningful to them.

*EM*, emergency medicine.
